# The biology of unconventional invasion of Duffy-negative reticulocytes by *Plasmodium vivax* and its implication in malaria epidemiology and public health

**DOI:** 10.1186/s12936-020-03372-9

**Published:** 2020-08-24

**Authors:** Lemu Golassa, Lucas Amenga-Etego, Eugenia Lo, Alfred Amambua-Ngwa

**Affiliations:** 1 Aklilu Lemma Institute of Pathobiology, Addis Ababa University, Addis Ababa, Ethiopia; 2 West African Center for Cell Biology of Infectious Pathogens, University of Ghana, Accra, Ghana; 3 Department of Biological Sciences, University of North Carolina at Charlotte, Charlotte, NC USA; 4 Medical Research Council Unit, The Gambia at London School of Hygiene and Tropical Medicine, Banjul, The Gambia

**Keywords:** *Plasmodium vivax*, Reticulocyte, Duffy antigens, Receptor, Ligand

## Abstract

*Plasmodium vivax* has been largely neglected over the past century, despite a widespread recognition of its burden across region where it is endemic. The parasite invades reticulocytes, employing the interaction between *Plasmodium vivax* Duffy binding protein (PvDBP) and human Duffy antigen receptor for chemokines (DARC). However, *P. vivax* has now been observed in Duffy-negative individuals, presenting a potentially serious public health problem as the majority of African populations are Duffy-negative. Invasion of Duffy-negative reticulocytes is suggested to be through duplication of the PvDBP and a novel protein encoded by *P. vivax* erythrocyte binding protein (EBP) genes. The emergence and spread of specific *P. vivax* strains with ability to invade Duffy-negative reticulocytes has, therefore, drawn substantial attention and further complicated the epidemiology and public health implication of vivax malaria. Given the right environment and vectorial capacity for transmission coupled with the parasite’s ability to invade Duffy-negative individuals, *P. vivax* could increase its epidemiological significance in Africa. In this review, authors present accruing knowledge on the paradigm shift in *P. vivax* invasion of Duffy-negative reticulocytes against the established mechanism of invading only Duffy-positive individuals and offer a perspective on the epidemiological diagnostic and public health implication in Africa.

## Background

Malaria affects one-third of the world’s population and kills hundreds of thousands across the globe [1]. *Plasmodium vivax* is the most widespread species of human malaria and more than 3 billion people live in *P*. *vivax*-endemic regions [2]. Unlike *Plasmodium falciparum, P. vivax* was believed not to be a major health problem in Africa. However, until recently, *P. vivax* infections have been widely reported in several African countries and its emergence is not surprising given the presence of a suitable environment, competent vectors and susceptible human hosts [3]. *Plasmodium vivax* not only differs from *P. falciparum* in terms of its epidemiology, but also its biology and evolutionary trajectory. These unique features enable *P. vivax* to survive under a wide range of climatic and ecological settings with the abilities to adapt to both temperate and tropical climates. Other features, including *P. vivax* ability to form hypnozoites, a stage of which the parasite can remain quiescent from months to years [4], an invasion preference of reticulocytes that only make up a small proportion of circulating erythrocytes, the absence of in vitro cultures, and rapidly forming gametocytes all together make elimination of vivax malaria more difficult than *P. falciparum* [5].

The epidemiology of *P. vivax* in Africa remains poorly understood, although *P. vivax* transmission from almost all countries across the continent has been reported from various entomological, serological, community prevalence surveys, including clinical infection data from local residents and travellers returning to malaria-free countries [6]. These reports have provided substantive evidence that *P. vivax* transmission is ongoing throughout Africa [3, 7]. However, there is still a paucity of data on the contemporary distribution of *P. vivax* in the region. There are increasing reports of *P. vivax* infections in Duffy-negative populations of Africa [8] as determined by different diagnostic tools. Indeed, *P. vivax* has been observed to be less severe in Duffy-negative Africans compared to their Duffy-positive counterparts [9]. This may be underpinned by the fact that *P. vivax* is poorly adapted to Duffy-negative individuals and may change as the parasite evolves to optimize its ability to infect Duffy-negative populations.

Here, authors review the contemporary biology and epidemiology of *P. vivax* among the spectrum of Duffy phenotype populations across Africa and synthesize data accrued on the invasion mechanisms of Duffy-negative erythrocytes and novel parasite ligands and host receptors that enable the parasites to invade reticulocytes. To achieve this, published articles was searched in the following electronic databases: Google Scholar, (www.google.scholar.com) and PubMed (www.pubmed.gov). Search terms included *Plasmodium vivax*, Duffy OR CD234 OR DARC, parasite ligands, host receptors, molecular epidemiology, invasion, mechanism. No restriction on year of publication was imposed but only articles published in English were considered.

Studies were selected if they met the following criteria: (1) The study involved human participants; (2) infections analysed were either *P. falciparum* and *P. vivax* co-infections or pure *P. vivax* infections; and, (3) studies were conducted in African or South American populations. Selected studies were entered into Mendeley Reference Manager and the following information was recorded: (1) country where study was conducted; (2) the case prevalence of *P. vivax* reported; (3) Duffy phenotypes reported; (4) comments on disease pathogenesis, particularly for Duffy-negative infections; (5) proposed mechanisms of infections of Duffy-negative individuals; (6) novel receptor–ligand interactions reported; and, (7) study conclusions.

### Diagnostic challenges associated with *Plasmodium vivax* infections

*Plasmodium vivax* infections persist with low levels of parasitaemia either as mono-infections or co-infections with *P. falciparum* in co-endemic settings, making it relatively difficult to diagnose with microscopy, which is the gold standard for malaria detection [10]. In most parts of Africa, particularly in Duffy-negative populations, malaria rapid diagnostic test kits (RDTs) are mainly aimed at detecting *P. falciparum* and sometimes minor species, such as *Plasmodium ovale* and *Plasmodium malariae,* but not specifically *P. vivax*, despite the increasing number of *P. vivax* reported across Africa and in Duffy-negative individuals. Therefore, *P. vivax* infections are often misdiagnosed either by mistake or because parasitaemia is too low given that its *P. vivax* invades reticulocytes that comprise only a small fraction of the circulating erythrocytes [5]. Owing to the increased ability to deploy molecular detection platforms, such as polymerase chain reaction (PCR), countries that had hitherto not reported any *P. vivax* infections are beginning to report increasing numbers of these infections in community surveys [11]. This is apparent that infections can be easily missed by the conventional microscopy and/or RDTs in such community-based surveillance [9, 12] and, therefore, hamper an accurate assessment of *P. vivax* prevalence in endemic areas across Africa. Moreover, with the advent of whole genome sequencing of *Plasmodium* spp, genome analysis of *P. vivax* from clinical sources will uncover the true magnitude of infections among Duffy-positive/negative populations [13].

### Evolutionary and clinical significance of *Plasmodium vivax* in Duffy-negatives

For many years epidemiologist thought that Duffy-negative individuals were totally resistant to vivax malaria and the complete resistance of Duffy-negatives to vivax malaria was the most striking example of association of a genetic trait with an infectious disease [14]. On the contrary, recent studies have demonstrated the capacity of *P. vivax* to infect human erythrocytes with or without the Duffy antigen to cause blood-stage infections [12, 15, 16]. A longitudinal study conducted in Mali by Niangaly et al. [17], found 2% asymptomatic *P. vivax* infections in Duffy-negative anaemic children. In another study conducted in Cameroon by Russo et al. [18], 27 *P. vivax*-infected Duffy-negative patients presented with fever. In admixture populations, according to Menard et al. [15], Duffy-positive individuals may serve as a reservoir of *P. vivax*, providing an opportunity for this parasite to infect hepatocytes of Duffy-negative people with the selection of novel *P. vivax* strains with the ability to invade Duffy-negative erythrocytes. Taken together, these data suggest a paradigm shift in the understanding of *P. vivax* invasion biology and raises more questions than answers. Could the adaptation of vivax to infect Duffy-negative individuals be frequency dependent? If so, what ratio of Duffy phenotypes fuel vivax adaptation to invade Duffy-negatives? There is yet limited evidence which supports the inability of vivax to spread to populations such as those in West Africa where the Duffy-negative allele is fixed. At any rate, given that *P. vivax* can cause both clinical and sub-clinical infections in Duffy-negative patients, these evolutionary realities are of public health concern, particularly when major investments in interventions against *P. falciparum* are driving falciparum to elimination but creating a niche for other *Plasmodium* spp.

### Epidemiology, disease severity and public health importance

While *P. falciparum* is considered the deadliest malaria parasite with most severe clinical outcomes, *P. vivax* is more widespread and often associated with high levels of morbidity. The overall burden, economic impact and severity associated with vivax malaria has been underestimated until some recent studies showing the association of *P. vivax* infections with severe malaria and death [19]. The clinical spectrum of *P. vivax*-associated malaria ranges from asymptomatic parasitaemia and uncomplicated febrile illness to severe and fatal malaria. Other severe clinical manifestations include multi-organ dysfunction with anaemia and thrombocytopaenia [20]. Another major public health concern of *P. vivax* is its association with spontaneous abortions, premature and low birth weight in pregnant women. These clinical features have mostly been described for Duffy-positive populations and may vary among Duffy-negative individuals in Africa. In areas where *P. falciparum* and *P. vivax* co-exist, while the total malaria burden decreases, the proportion of cases attributable to *P. vivax* increases [21].

In *P. vivax*, gametocytes appear simultaneously with the asexual schizonts [22] while in *P. falciparum*, gametocytes usually appear 10–14 days after infection. Such a difference in development not only increases the length of time the individual remains infectious, but also increases the likelihood of transmission before the infected individual seeks treatment [23]. Moreover, *P. vivax* ability to relapse from dormant liver-stage hypnozoites, from weeks to years after clearance of the primary blood-stage infection is a major obstacle to its control and elimination [24]. The increased population at risk of *P. vivax* infection and the growing clinical burden across regions with a spectrum of Duffy phenotypes puts vivax malaria on the spotlight as an important public health issue [25]. Definitively, Duffy-negative phenotype is not a barrier to *P. vivax* infection and the public health significance of vivax malaria should no longer be neglected [25].

### *Plasmodium vivax* tropism to reticulocytes and Duffy antigens

The sparse distribution of *P. vivax* in Africa is viewed as a consequence of the lack of expression of the Duffy antigen on red blood cells (RBCs). Exhibiting distinct red cell tropism, malaria parasites use sophisticated invasion mechanisms to identify, penetrate and multiply in vector and human host cells. *Plasmodium vivax* blood-stage infection is restricted to human reticulocytes, whereas *P. falciparum* can infect all types of RBCs [26]. RBC invasion by *Plasmodium* species requires multiple interactions between parasite ligands and host receptors, some of which have overlapping and partially redundant roles [27]. With the evolutionary arms race between the malaria parasites and human hosts, variants of erythrocyte receptors and parasites ligands have evolved. In *P. vivax,* the connection between the *Pv*DBP and DARC is a critical pathway, and the only pathway identified so far, for *P. vivax* invasion of human reticulocytes, in spite of the increasing reports of *P*. *vivax* infections in Duffy-negative individuals that rise questions on the specificity of Duffy antigens [28, 29]. With increasingly reports of *P. vivax* infection in Duffy-negative populations throughout Africa and South America, it is possible that these infections were historically not detected rather than a recent adaptation of certain *P. vivax* strains.

The invasion of human reticulocytes by *P*. *vivax* had been known to be completely dependent on the interaction between PvDBP and DARC [30], unlike *P*. *falciparum* merozoites that use several erythrocyte receptors (e.g., glycophorin A (GPA) and glycophorin C (GPC) and ligands (e.g., EBA-175 and EBA-140) for erythrocyte invasion. Although DARC is present on the erythrocyte surface, *P. vivax* reticulocyte binding proteins (PvRBPs) bind receptors on reticulocytes that are subsequently lost during erythrocyte maturation [31]. This enhances the exposure of PvDBP binding pocket to young reticulocytes explaining *P. vivax* tropism [32]. Based on sequence homology, the PvRBP family comprises PvRBP1 (composed of PvRBP1a and PvRBP1b) and PvRBP2 (composed of PvRBP2a, PvRBP2b, PvRBP2c) [33]. PvRBP2d and PvRBP3 are pseudo-genes that share homology with PvRBPs but do not encode functional proteins. According to Miller et al., Duffy-negative individuals can be infected by *P. vivax*, but are protected from blood-stage *P. vivax* infection [34] indicating that such infections could persist as asymptomatic.

### DARC polymorphisms and *Plasmodium vivax* invasion canon

DARC is a membrane protein present on the surface of erythrocytes and central to *P. vivax* merozoites invasion of RBCs. Due to its strong geographic differentiation and association with vivax malaria resistance, DARC is considered as best example to depict positive selection in human genome. It belongs to the G protein-coupled receptor family (a 35–50 kDa glycoprotein) expressed both on the surface of RBCs and endothelial tissue [35]. Duffy proteins are also expressed in erythroid precursor cells of the bone marrow where *P. vivax* invasion could be occurring to some degree [26]. The Duffy glycoprotein is encoded by the *FY* gene main alleles, FY*A (FY*01) and FY*B (FY*02) that are codominantly expressed (Table 1). Each allele can be inherited either from mother or father and both gene products, Duffy Fy^a^ and Fy^b^ antigens, can be expressed on the RBCs. Fy^a^ and Fy^b^ differ by a single amino acid at position 42 that encodes glycine in Fy^a^ and aspartic acid in Fy^b^. Antibodies against Fy^a^ and Fy^b^ (anti-Fy^a^ and anti-Fy^b^) define four main phenotypes: Fy(a+b−), Fy(a+b+), Fy(a−b+) and Fy(a−b−). The Duffy-negative phenotype, Fy(a−b−), occurs in two-thirds of African–American Blacks [36] and is associated with a single point mutation c.1-67T>C (rs2814778) in the GATA-1 binding motif for the erythroid promoter of FY*B  [35] that prevents the expression of FY*B in RBCs (thus blocks the RBC invasion by *P. vivax*), but without affecting FY*B expression in other tissues [37, 38].

**Table 1:** Variants of the Duffy blood group (ISBY008)

Phenotype	Allele name	Nucleotide change^a^	Exon	Predicted amino acid change
FY:1 or Fy(a+)	FY*01 or FY*A	c.125A>G	2	p.Asp42Gly
FY:2 or Fy(b+)^b^	FY*02 or FY*B			
Null phenotypes				
Fy(a−b−) erythroid cells only	FY*01N.01	c.-67T>C	Promoter	p.0
Fy(a−b−)	FY*01N.02	c.281_295del	2	p.Pro94_Val98del
Fy(a−b−)	FY*01N.03	c.408G>A	2	p.Trp136Ter
Fy(a−b−)	FY*01N.04	c.287G>A	2	p.Trp96Ter
Fy(a−b−)	FY*01N.05	c.327delC	2	p.Phe109Leufs*12
Fy(a−b−)	FY*01N.06	c.395G>A	2	p.Gly132Asp
Fy(a−b−)	FY*01N.07	c.719delG	2	p.Gly240Alafs*4
Fy(a−b−) erythroid cells only	FY*01N.08	c.-69T>C	Promoter	p.0
Fy(a−b−)	FY*01N.09	c.296_496delinsAGGCCACTG	2	p.Leu99_Leu165delins GlnAlaThrAla
Fy(a−b−) erythroid cells only	FY*02N.01	c.-67T>C	Promoter	p.0
Fy(a−b−)	FY*02N.02	c.407G>A	2	p.Trp136Ter
Fy(a−b−)	FY*02N.03	c.781G>A	2	p.Gly261Arg
Fy(a−b−)	FY*02N.04	c.179_180delCT	2	p.Ser60Cysfs*16
Fy(a−b−)	FY*02N.05	c.895G>A	2	p.Ala299Thr
Fy(a−b−)	FY*02N.06	c.151delT	2	p.Cys51Alafs*24
Weak phenotypes				
Fy(a+w)	FY*01W.01	c.265C>T	2	p.Arg89Cys
Fy(a+w)	FY*01W.02	c.265C>T	2	p.Arg89Cys
		c.298G>A		p.Ala100Thr
Fy(a+w)	FY*01W.03	c.680G>A	2	p.Gly227Glu
Fy(b+w), Fyx	FY*02W.01	c.265C>T	2	p.Arg89Cys
		c.298G>A		p.Ala100Thr
Fy(b+w), Fyx	FY*02W.02	c.145G>T	2	p.Ala49Ser
		c.265C>T		p.Arg89Cys
		c.298G>A		p.Ala100Thr
Fy(b+w)	FY*02W.03	c.266G>A	2	p.Arg89His
Fy(b+w)	FY*02W.04	c.901C>T	2	p.Pro301Ser

^a^ Nucleotide numbering within the transcript is numbered according to the major transcript. The GATA-1 mutation listed here as c.-67T>C has been reported previously as − 33 and − 46

^b^ Reference allele FY*02 encodes FY3, FY5, FY6

The gene encoding the Duffy blood group, *Fy*, is characterized by a SNP in a GATA-1 transcription factor binding site associated with the erythrocyte silent (ES) phenotype that has been shown to protect against *P. vivax* infection and is at near fixation in sub-Saharan Africa and is virtually absent any where else. Indeed, the Duffy antigen exhibits extreme geographic differentiation in Africa, but nearly absent from Asia and Europe. While ES (Fy*B^*ES*^ allele; FY*02N.01) phenotype impairs promoter activity in erythroid cells by disrupting a binding site for the GATA-1 erythroid transcription factor [37] and confers complete protection from vivax malaria, low levels of *P. vivax* infections have been observed in Fy*01N.01 homozygotes [39], which indicates that *P. vivax* might be evolving escape-variants able to overcome the protective effect of Fy*01N.01/FY*01N.01 or has evolved a *Fy*-independent RBC invasion pathway or that the GATA-1 SNP does not abolish *Fy* expression. Different allelic forms of the Duffy blood group gene could also modify the antigen’s expression level, leading to weak phenotype. For example, two variants c.265C>T (rs34599082) and c.298G>A (rs13962) of glycoprotein Duffy have resulted in weak Fy^b^ expression [40]. Another mutation c.145G>T generating the FY*02W.02 allele has also shown to weaken the expression of the Fy^b^ antigen [41, 42]. The expression level of these different weak phenotypes needs verification through flow cytometry in future studies.

### *Plasmodium vivax* DBP gene duplication and Duffy-negative reticulocyte invasion

Red blood cell invasion by *P*. *vivax* merozoites appears to rely heavily on the interaction between PvDBP and DARC [43]. Whole-genome sequencing of *P. vivax* clinical isolates collected from Duffy-negative individuals indicates the presence of two and higher copies of the PvDBP gene [44]. This observation suggests that the PvDBP gene is under strong positive selection [45]. Two different types of the duplication have been observed among *P. vivax* clinical isolates from Cambodia [46], Ethiopia [44] and Madagascar [47]. Moreover, analysis of *P*. *vivax* genomes from over 200 isolates originating from across the world indicated that copy number variation (CNV) at the PvDBP locus is one of the most common CNVs within the *P*. *vivax* genome irrespective of the geographical origin of the isolates [13]. For instance, in Cambodia, where Duffy-negativity is essentially absent, the frequency of PvDBP duplication had reached 40% [13]. PvDBP CNVs seem to be continuously arising de novo, occurring independently within defined geographic boundaries based on phylogeographic analysis. Apparently, PvDBP is also evolving rapidly among Duffy-negative population in most parts of the sub-Saharan Africa, presumed to be the origin of *P. vivax* [48]. In other geographic areas where almost all individuals are Duffy-positives, PvDBP duplications have also been observed with high frequency [13]. The outcome of this repeated gene duplications (or by population size expansion) from the ancestral lineage in *P. vivax* indicates that several sub-populations are present [49].

CNVs are common in both *P. falciparum* and *P*. *vivax* [13]. What initiates PvDBP expansion in *P. vivax*? For example, expansion of *mdr*1 has been shown to result in increased anti-malarial resistance, however, it is unlikely that anti-malarial drug pressure plays a role in PvDBP duplication unless the duplication confers an increased growth rate to *P.* *vivax* parasites [44]. Owing to the critical role that PvDBP-DARC interaction plays in *P*. *vivax* erythrocyte invasion, it is expected that the binding affinity of DARC with high-copies PvDBP could be higher than with single-copy PvDBP parasites. However, no association has been found between PvDBP duplications and the efficiency of parasite to invade reticulocytes with FY*A or FY*B genotypes [44]. Assuming a high adaptability of *Plasmodium* species, it is likely that the duplications emerged in the parasites in response to the variation in human Duffy blood group across malaria-endemic settings. Recent studies have shown that PvDBP gene amplification not only facilitates binding to an alternative lower affinity receptor in Duffy-negative reticulocytes [16, 44], but also allows *P. vivax* to evade host anti-PvDBP humoral immunity [50], reassuring PvDBPII region as a promising candidate for a blood-stage vaccine against *P. vivax* [34]. The polymorphic nature of *Pv*DBP certainly allows *P. vivax* to colonize diverse ecological niches as well as to evade the host immune system.

A molecular epidemiology study conducted in Ethiopia observed that the proportion of parasites with PvDBP amplification was higher in individuals carrying the FY*A allele compared to individuals carrying the FY*B [51]. On the other hand, in Madagascar where both Duffy-negative and Duffy-positive individuals coexist, PvDBP amplification is not selected in response to the Duffy null homozygotes [52]. It is noteworthy to see a higher frequency (56%) of multiple PvEBP copies in Madagascar where Duffy-negative and Duffy-positive people coexist than in Cambodia (19%) where mostly Duffy-positive people are present [53]. Such a contrast may imply a functional role of PvEBP amplification towards Duffy-negative erythrocyte invasion.

### Evidences of *Plasmodium vivax* in Duffy-negative individuals

The first indirect evidence of invasion of Duffy-negative reticulocytes by *P. vivax* came from travellers who presented with *P. vivax* infection after returning from countries in Africa where Duffy-negativity is at near fixation [54, 55]. Because *P. vivax* can remain as latent infections for months or even years before triggering a relapse infection, it is difficult to confidently identify the source of *P. vivax* responsible for the blood stage infection in those individuals. In addition, there is yet insufficient data demonstrating the presence of *P. vivax* in *Anopheles* mosquitoes collected in areas where the majority of people are Duffy-negative [39]. However, with the advent of sensitive molecular detection techniques, there has been a growing number of reports of *P. vivax* infections in Duffy-negative individuals.

The detection of *P. vivax* DNA by molecular and serological assays in a Duffy-negative individual’s blood sample could represent pre-erythrocytic stages of the parasites, independent of blood-stage infection [48]. Therefore, microscopic observation of *P. vivax* within Duffy-negative erythrocytes coupled by confirmation by genotyping has essentially proved Duffy-negative infection [50]. The first microscopic observation of *P. vivax* infection in a Duffy-negative erythrocyte was reported by Ryan et al., in Kenya in [39] followed by a definitive evidence of a *P. vivax* infection within Duffy-negative erythrocyte from patients in Madagascar [15]. Further experimental evidence on host susceptibility was obtained from a Nigerian who got infected with *P. vivax* through mosquito bite followed by the establishment of *P. vivax* blood-stage infection [55], though the Duffy status of the infected individual was not confirmed. Future investigations of expanded anti-*P. vivax* antibody testings in Duffy-negative populations would reveal the extent of exposure and possible adaption of *P. vivax* for continuous transmission in these populations. Furthermore, *P. vivax* infections of Duffy-negative people have also been reported from the American continent using serological, molecular, and microscopic detection tools [56, 57]. While Duffy negativity is clearly no longer a barrier against *P. vivax* infections, a recent study of *P. vivax* patients in the Brazilian Amazon region has shown that the null allele FY*02N.01 and weak allele FY*02W.01 were associated with low parasitaemia and low symptomatology [58]. The presence of the polymorphic alleles could lead to a possible reduction in *P. vivax* adhesion by the reduction of the Duffy glycoprotein, as supported by earlier studies showing erythrocytes, expressing the FY*/FY*02N.01, have a significant reduction in parasite adhesion when compared to erythrocytes expressing FY*02/FY*02 [59–61]. Therefore, the presence of FY*02N.01 and FY*02W.01 alleles may have an impact on the reduction of clinical manifestation in malaria, leading to the development of sub-clinical malaria.

### Suggested mechanisms of Duffy-negative reticulocyte invasion by *Plasmodium vivax*

Understanding of the mechanisms of RBC invasion by *P. vivax* in the absence of Duffy-positive receptor is imperative with respect to parasite epidemiology, public health importance and elimination strategies against vivax malaria. To date, the question of whether *P. vivax* has recently evolved its strategies to infect Duffy-negative erythrocytes and utilized an alternative DARC-independent invasion pathway remains unclear. Characterizing this pathway is important in the development of innovative strategies to prevent spread of *P. vivax* infections in Africa, and vaccine development. Unfortunately, the inherent challenge of culturing *P. vivax* in vitro has made studies targeting vivax malaria very difficult [62]. The involvement of putative parasite ligands in the invasion of Duffy-negative individuals has been described in a number of studies, but it remains unclear what specific receptor on the human erythrocyte is aiding the entry of *P. vivax* in Duffy-negative reticulocytes [50].

**Fig. 1:**
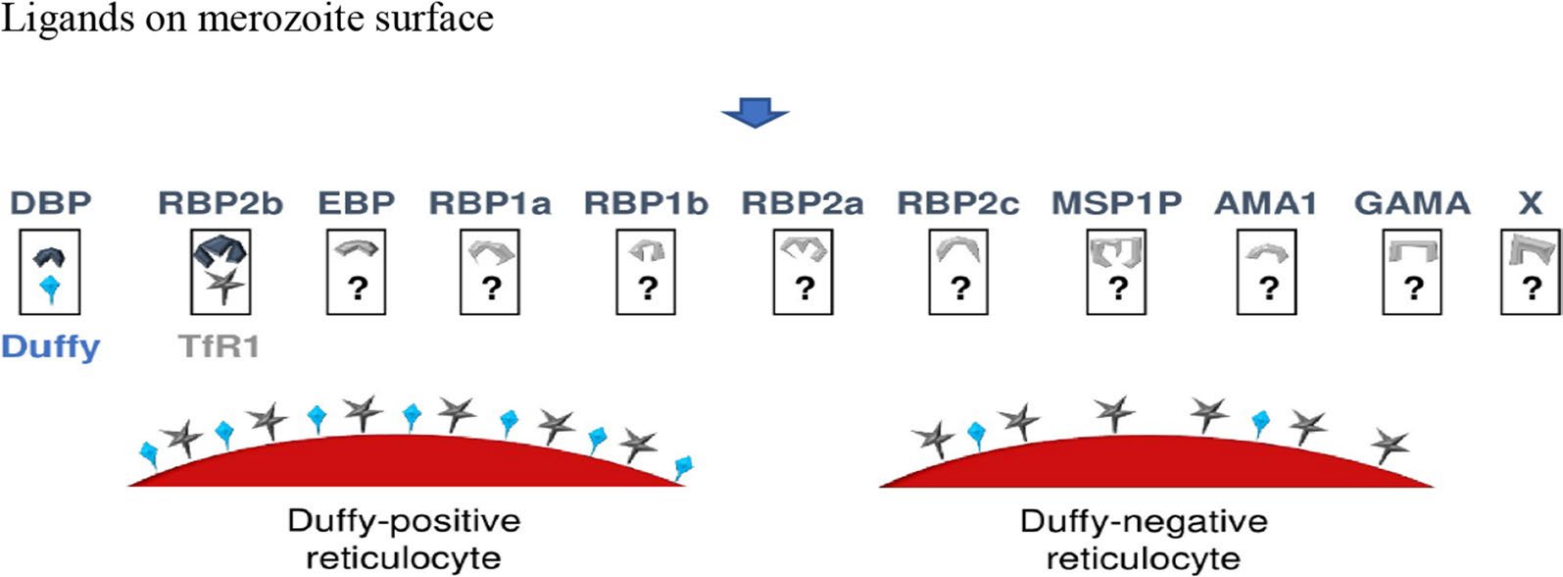
Interaction between parasite ligands and reticulocyte receptors during invasion process of Duffy-positive and Duffy-negative cells by *Plasmodium vivax* (adapted from Popovici et al. [50]). A spectrum of ligands on the merozoite surface of the parasites that may bind to Duffy-negative and Duffy-positive reticulocytes are presented

One suggested alternate invasion pathway of *P. vivax* is through the interaction between the human Duffy antigen/receptor for chemokines (DARC, CD234) and the *P. vivax EBP*2, a new member of the DBP family [63]. Based on genome sequences of *P. vivax* field isolates and monkey-adapted strains, two possible invasion mechanisms have been proposed. The first mechanism is through duplication of the PvDBP gene observed in multiple *P. vivax* strains. The other is invasion through a novel PvEBP-driven pathway that contains conserved Duffy-binding-like and C-terminal cysteine-rich domains that can be recognized by the Duffy receptor (Fig. 1) [64]. It is unclear what other possible host receptor(s) are recognized by PvEBP during the invasion process in Duffy-negative individuals. However, panels of receptors on erythrocytes and reticulocytes as well as parasite ligands have been well described as the possible alternative pathways for Duffy-negative invasion [9].

One potential parasite ligands involved in Duffy-positive/Duffy-negative reticulocyte invasion is the family of *P. vivax* reticulocyte binding proteins (PvRBPs) [31]. Although PvRBPs ligands bind to erythrocytes, there are contradictory results regarding their specific binding to reticulocytes [9, 65]. Of the different *Pv*RBPs previously examined, PvRBP1a and PvRBP2c bind to Duffy-negative erythrocytes [31]. However, PvRBP1a does not seem to be critical for *P. vivax* invasion of Duffy-positive cells [66]. The third member of the *Pv*RBP family, PvRBP2b, was recently shown to be a key ligand involved in reticulocyte recognition and invasion through the transferrin receptor 1 (TfR1) [29]. However, the role of PvRBP1a and of PvRBP2b-TfR1 recognition of Duffy-negative red blood in *P. viv*ax invasion of reticulocytes remain unclear. The membrane protein TfR1 is lost during red blood cell maturation and, therefore, absent in normocytes [67]. Theoretically, TfR1 should be present on the surface of both Duffy-positive and Duffy-negative reticulocytes. The critical interaction between PvRBP2b and TfR1 occurs upstream of the PvDBP-Duffy interaction, but is not independent of the latter [29]. Although the role of this interaction in Duffy-negative has not been assessed, it is proposed to play a key role in Duffy-independent invasion pathways [50]. PvRBP2b first binds to the TfR1 present on reticulocytes before Pv*DBP* engages with the Duffy protein, allowing entry of the merozoite in the red blood cell during invasion in Duffy-positives [50]. It has been shown that DARC knockout and TfR mutants individually reduced *P. vivax* parasite invasion.

*Plasmodium vivax* merozoite surface protein-1 paralog (PvMSP1P) and *P. vivax* glycosylphosphatidylinositol-anchored micronemal antigen (PvGAMA) were also shown to bind to both Duffy-positive and Duffy-negative RBCs [68–70]. Taken together, this suggest that PvGAMA and PvMSP1P ligands could be involved in Duffy-independent reticulocyte invasion pathways even though their function is yet to be fully determined. Furthermore, *P. vivax* erythrocyte-binding protein (PvEBP, also called PvDBP2) has also been shown to bind moderately to Duffy-negative reticulocytes [64, 71].

It is as yet unclear whether a few Duffy molecules present on the surface of the erythrocyte enabling parasites with multiple PvDBP gene copies to invade the cell, or whether the invasion process of Duffy-negative reticulocytes could occur through alternate pathways by non-DBP ligands (PvEBP, PvMSP1P, or PvGAMA) [50]. Another plausible explanation for Duffy-negative invasion is that the Duffy-negative phenotype may not be entirely a Duffy null and that the Duffy receptor may be expressed at a subtle level sufficient enough for low level invasion. The c.-67T>C mutation in GATA-1 transcription factor has been confirmed in *P. vivax*-infected Duffy-negative individual [15]. This mutation is known to reduce the binding of the GATA-1 transcription factor and gene transcription. However, the reduced levels of RNA transcripts may still permit low expression and binding of the Duffy receptor protein with PvDBP. Thus, it is possible that Duffy-negative individuals could have been asymptomatic carriers of *P. vivax* for some time before symptoms emerged. These asymptomatic cases could have escaped the detection by current diagnostic tools and/or regimes in most African countries that primarily target *P. falciparum*. *Plasmodium vivax* is now confirmed not to be completely absent from western and central Africa, though the dominance of Duffy-negative individuals in these regions has kept the prevalence of *P. vivax* low. Moreover, the presence of very low proportion of Duffy-positive individuals could have minimized *P. vivax* transmission in these regions, though there is no evidence of direct transmission among Duffy-negative individuals and between Duffy-positive and Duffy-negative individuals. Lastly, the contribution of alternative hosts such as apes [72], the geographical range of *P. vivax* parasites [73], its red blood cell invasion protein repertoire [74], and the human and non-human primate hosts that are infected [75] cannot be neglected in facilitating transmission. Given that Duffy-negative people are not completely resistant to *P. vivax* blood stage malaria [11], new reservoirs of *P. vivax* within infected individuals must be considered [26].

## Conclusion

Infection of Duffy-negative individuals by *P. vivax* remains an enigma for parasitology and evolution. Since most individuals of African origin are Duffy-negative and do not express the DARC antigen on the surface of their erythrocytes, it is unclear whether the high prevalence of Duffy-negative alleles in African populations results from *P. vivax* resistance selection [76] or there exists hidden transmission of *P. vivax* among Duffy-negative populations. The public health significance of *P. vivax* is increasingly apparent in Africa. Duffy-negative reticulocyte infection by *P. vivax* highlights the risk of emergence and spread of vivax malaria in Africa where it was non-existent before. Apart from the Horn of Africa, Madagascar and Mauritania, *P. vivax* is grossly overlooked in many parts of the African continent. Given the low parasitaemia associated with *P. vivax* infections in Duffy-negative individuals, microscopy and RDTs are not sensitive enough to detect. Therefore, the contemporary epidemiology of vivax malaria in Africa is vastly unknown [17]. This calls for sensitive molecular detection tools such as PCR for the diagnosis of low-density *P. vivax* infections and studies that further characterize genomic loci under selection in *P. vivax* isolates infecting Duffy-negative individuals. Future studies towards a better insight of erythrocyte invasion mechanism could be obtained by comparison of gene expression profiles of parasites infecting Duffy-negative and Duffy-positive people and identifying genes specifically up-regulated in Duffy-negativity infections. The evolution of *P. vivax* strains able to infect RBCs not expressing Duffy antigen could have important implications for vaccine development. Increasing efforts for continuous surveillance of *P. vivax* strains capable of invading red cell through Duffy-independent pathway is essential for designing *P. vivax*-specific vaccine candidates. A better understanding of the epidemiology of *P. vivax* enables the design of *P. vivax*-specific control and elimination strategies. Given the presence of an ideal temperature, highly competent vectors for its transmission, and the apparent increase in the host range, *P. vivax* seems to expand to areas where it was non-existent in the past in Africa. It is imperative to consider how the growing evidence that *P. vivax* is not restricted to a Duffy-dependent invasion pathway is affecting the African population at risk of vivax malaria.

## Data Availability

Not applicable.

## Abbreviations

CNVs: Copy number variants
GPA: Glycophorin A
GPC: Glycophorin C
DARC: Duffy antigen receptor for chemokines
MAP: Malaria Atlas Project
MDR1: Multidrug resistance 1
SNP: Single nucleotide polymorphism
PCR: Polymerase chain reaction
PvDBP: *Plasmodium vivax* Duffy binding protein
PvEBP: *Plasmodium vivax* erythrocyte binding protein
PvGAMA: *P. vivax* glycosylphosphatidylinositol-anchored micronemal antigen
PvMSP1P: *Plasmodium vivax* merozoite surface protein-1 paralog
PvRBPs: *Plasmodium vivax* reticulocyte binding proteins
RNA: Ribonucleic acid
RDTs: Malaria Rapid diagnostics tests
RBPs: Reticulocyte binding proteins
RBCs: Red blood cells
TfR: Transferrin receptor
